# Age-related changes in visual exploratory behavior in a natural scene setting

**DOI:** 10.3389/fpsyg.2013.00339

**Published:** 2013-06-21

**Authors:** Johanna Hamel, Sophie De Beukelaer, Antje Kraft, Sven Ohl, Heinrich J. Audebert, Stephan A. Brandt

**Affiliations:** ^1^Department of Neurology, University of Rochester Medical Center, University of RochesterRochester, NY, USA; ^2^Department of Neurology, Charité Campus Mitte, Universitätsmedizin CharitéBerlin, Germany; ^3^Berlin School of Mind and Brain, Humboldt Universität zu BerlinBerlin, Germany; ^4^Department of Neurology, Charité Campus Benjamin Franklin, Universitätsmedizin CharitéBerlin, Germany; ^5^Center for Stroke Research Berlin, Universitätsmedizin CharitéBerlin, Germany

**Keywords:** oculomotor behavior, eye movements, visual attention, driving simulator, aging, video game experience

## Abstract

Diverse cognitive functions decline with increasing age, including the ability to process central and peripheral visual information in a laboratory testing situation (useful visual field of view). To investigate whether and how this influences activities of daily life, we studied age-related changes in visual exploratory behavior in a natural scene setting: a driving simulator paradigm of variable complexity was tested in subjects of varying ages with simultaneous eye- and head-movement recordings via a head-mounted camera. Detection and reaction times were also measured by visual fixation and manual reaction. We considered video computer game experience as a possible influence on performance. Data of 73 participants of varying ages were analyzed, driving two different courses. We analyzed the influence of route difficulty level, age, and eccentricity of test stimuli on oculomotor and driving behavior parameters. No significant age effects were found regarding saccadic parameters. In the older subjects head-movements increasingly contributed to gaze amplitude. More demanding courses and more peripheral stimuli locations induced longer reaction times in all age groups. Deterioration of the functionally useful visual field of view with increasing age was not suggested in our study group. However, video game-experienced subjects revealed larger saccade amplitudes and a broader distribution of fixations on the screen. They reacted faster to peripheral objects suggesting the notion of a general detection task rather than perceiving driving as a central task. As the video game-experienced population consisted of younger subjects, our study indicates that effects due to video game experience can easily be misinterpreted as age effects if not accounted for. We therefore view it as essential to consider video game experience in all testing methods using virtual media.

## Introduction

It is widely accepted that as we age, cognitive function becomes increasingly impaired as part of the normal aging process (Park et al., 2003; Hedden and Gabrieli, 2004; Morrison and Baxter, 2012). Recent research efforts have focused their interest on the changes in higher cognitive functions such as memory systems (Podell et al., 2012) or on age-related changes in sensory functions, which represent an earlier step in cognitive processing (Cliff et al., 2012; Mozolic et al., 2012). In the visual domain, recent studies have addressed the hypothesis that visual perception deteriorates with increasing age: to test this, Owsley et al. (1998) developed a model of the “Useful Field of View” (UFOV), referring to the spatial area from which a person is able to simultaneously process central and peripheral visual information. The UFOV is measured by cognitive tests that are sensitive to declines in visual sensory function, slowed visual processing speed, and impaired visual attention skills.

It is of great interest how these laboratory test results correlate with relevant changes in daily life activities. While driving a vehicle for example the information processed is mainly visual (Robinson et al., 1972). Studies have ascribed a greater risk of causing road traffic accidents (Tefft, 2008) to older driving populations.

How does visual processing while driving change with age and how does this alter driving behavior? To address this question we set up a study to investigate age-related changes in visual exploratory behavior in a natural setting: we used a driving simulator with subjects driving routes of varying complexity while simultaneously wearing a head-mounted camera recording eye- and head-movements, as well as measuring reaction times (screenshot of driving course, Hamel et al., 2012).

It has been previously shown that UFOV parameters have a high sensitivity (89%) and specificity (81%) in predicting which older drivers have a history of car crashes (Ball et al., 1993). A prospective study demonstrated that older drivers with a 40% or greater impairment in the UFOV were more than two times more likely to cause a crash during a 3-year follow-up period (Owsley et al., 1998).

Two different theories were developed of how the peripheral field deteriorates in a realistic setting, e.g., a driving situation, while central demand increases: either through an abrupt neglect of stimuli, referred to as general interference (Holmes et al., 1977; Crundall et al., 1999) or through gradual decline of attention with increasing eccentricity, referred to as tunnel vision (Williams, 1988; Miura, 1990). Recent studies demonstrated a combination of both in older drivers: while presenting stimuli not related to the driving task, tunnel vision was observed, whereas if stimuli were relevant to driving (e.g., because they were located on other vehicles), a general interference occurred (Rogé et al., 2004). In our study we used targets located at different eccentricities, but all relevant to the driving task.

Besides peripheral visual perception, gaze behavior also contributes to the acquisition of visual information in natural activities such as driving. To this end, an important approach was introduced by Mourant and Rockwell (1972). They demonstrated that fixations and saccadic eye movements provide important insights into the drivers' visual search behavior, information needs, and information-acquisition processes (Shinar, 2008). They showed that search and scan patterns of novice drivers differed from those of experienced drivers as they covered a smaller visual field, which was interpreted as unskilled and inadequate for the detection of potential hazards. However, not only eye movements give an insight into exploratory behavior, head-movements have become the focus of research interest in naturalistic tasks, and the role of age-dependent changes in head-movement-behavior remains unclear. Our study allows for and examines unrestrained movements of the head, as there is evidence supporting an increased number of head-movements among the elderly as a compensatory strategy in visual tasks (Proudlock et al., 2004). Also, in head-unrestrained conditions, eye movement characteristics change, depending on gaze amplitudes (Freedman, 2008).

A high variability in visual search behavior has been demonstrated among the elderly (Maltz and Shinar, 1999). During episodes in which the older participants had difficulty searching, their eye movements were characterized by shorter saccades and increased numbers of fixations. Other studies (Pradhan et al., 2005; Bao and Boyle, 2009) showed that older drivers scanned significantly less toward both sides when passing intersections compared to middle-aged and younger drivers and searched less often for possible hazards than younger drivers (Romoser and Fisher, 2009). This was interpreted as indicating visual inattention, e.g., in the form of (a) a poor visual search process with ineffective use of the peripheral field, or (b) a failure to extract maximum amounts of information from areas that were already fixated on (Maltz and Shinar, 1999).

In order to characterize possible visual inattention, one goal of the study is to describe visual information-acquisition skills in more detail: we differentiated between manual reaction times, which could be delayed by motor impairment, and the latency until an object or hazard is fixated by the driver and possibly seen. Manual (Crundall et al., 2002; Rogé et al., 2004; Jahn et al., 2005) or verbal (Cantin et al., 2009) reaction times have been chosen by previous studies as a response. If there was a difference between the two, it would not only be of diagnostic interest, but also training could be specified to improve manual reaction time either by raising more attention, or by training the pure motor responses. Observation of gaze fixations of risk relevant elements rather than manual reaction times was used to examine risk recognition in novice, young experienced, and older drivers (Pradhan et al., 2005). Overall, 25.82% of the younger, novice drivers, 40.14% of the younger, experienced drivers, and surprisingly 69.59% of the older drivers demonstrated visual exploratory behavior which indicated that they recognized the risks in a scenario. Nevertheless, the fatality rate of the older drivers was not smaller than that of the younger drivers, although the older drivers showed more awareness of the risks. The authors argued that the increasing risk awareness compensates for changes in the cognitive, perceptual, and motor status.

Interestingly, in a training session including a feedback mechanism displaying the participants point of gaze, older drivers could be successfully taught to perform more second looks for potential hazards (Pollatsek et al., 2012). Pollatsek argues that these findings may be explained by the following: older drivers develop an “unsafe habit,” which can be reversed by training, rather than deteriorating physical or mental capabilities. Thus, training may be effective in reducing crashes. The unsafe habit could consist of the goal not to hit anything that is in front of their vehicle, similar to a central driving task. In this study, examining the gaze distribution and reaction times via fixation will investigate whether elderly drivers tend toward a central fixation bias.

Comparability of studies with each other and with actual driving situations is limited: Pollatsek offered stable pictures of traffic situations, which were explored and did not include a dynamic testing situation. In some studies head-movements were not measured (Pradhan et al., 2005) and eye movements were recorded via video recording instead using an eyetracker (Bao and Boyle, 2009). Another factor, which we accounted for in our study, is the experience of participants with video or computer games, which presents a potential confounder and major effect on study results. In previous studies testing spatial memory in a virtual driving task with pointing and navigating, virtual media experience was associated with greater pointing accuracy and greater navigational efficiency (Han et al., 2012). The authors argued that one reason for this pattern of results could be that the advantage conferred by video game experience in the task is due to better spatial encoding and recall of routes. It has been shown that video game-experienced subjects more successfully suppress distracting information and reveal enhanced attention to objects (Bavelier et al., 2011). They also reveal better performance in allocating spatial attention over the visual field at all eccentricities (Green and Bavelier, 2003). Action video game players tend to employ efficient executive strategies to reduce distraction during tasks (Chisholm and Kingstone, 2012). Video game interventions have offered some promise to help with cognitive decline in the elderly population. However, its mechanism and successful use remains unclear (Boot et al., 2013).

## Materials and methods

### Participants

Eighty-five participants were recruited (by newspaper advertisement, a local senior computer club, and among hospital employes) of varying age (20–75 years of age, equally distributed). The study was conducted in conformity with the declaration of Helsinki and was approved by the local ethics committee. Written informed consent was obtained from all participants. All subjects were paid for their participation and were unaware of the purpose of the experiments. In the first session a medical history was taken and experiences with virtual media explored. All participants reported no cognitive deficits, neurological or psychiatric deficits or diseases, and visual acuity was higher than 0.5. Seven participants did not own a driver's license. One subject did not attend the second test day, 11 participants had to be excluded from analysis [due to strabismus (2), ptosis, multiple blinks, or difficulty in calibration (3), malposition of the head (2), or nausea (4)]. The remaining 73 participants fully completed all testing sessions and were included in the analysis.

### Experimental setup

Participants were tested in a fixed base driving simulator (Hamel et al., 2012). The simulator consisted of a simulation car seat imitating a real car seat (including brake and accelerator pedals, steering wheel, and indicators). A special software (SILAB 3.0 by Wuerzburg Institute for Traffic Sciences GmbH (WIVW)] was used to program specific driving scenarios meeting the requirements of the study by providing courses with different amount of workload and recording the driver's performance.

The software was combined with a head-mounted binocular infrared video pupil tracker, recording head- and eye-movements at a sample rate of 100 Hz (EyeSeeCam by University of Munich Hospital, Clinical Neurosciences). The eye data were calibrated using a series of five fixation points.

A projector (Canon SX 80 Mark II, resolution: 1400 × 1050 pixels) displayed the visual information on a screen (1.52 m high × 2.03 m wide) located 2.0 m from the participant's head. The center of the screen was located at eye level through the mid-line of the subject, thus covering 58.15° of the visual angle on the horizontal axis and 43.61° on the vertical axis of the field of view.

### Driving task

At the first session, instructions were given regarding the driving task and how to use the simulation vehicle. A practice run with less task density was conducted to allow familiarization to the simulation situation, the task and to prevent simulator sickness, a syndrome similar to motion sickness potentially confounding data and increasing the drop-out rate (Brooks et al., 2010). Prior to the start participants were informed that the simulator could cause experiencing nausea. They were specifically instructed to inform the experimenter if this happened and were told the experiment would stop immediately with no consequences. A rest of at least 1 day was assured between the practice and the experimental run. About 30% of the participants, when specifically asked after the completion of the experiment, reported retrospectively feeling slight malaise during the testing session.

The participants accomplished two experimental runs. Each consisted of a continuous scenario of 6500 m of rural roads (approximately 10 min duration) and different task difficulty due to level of distraction by surrounding environment (easy vs. difficult). Both drives were equal in setup and located in a rural scene. The easy course had lower stimulus density (referred to as “field”), with flat farmed fields allowing for clear visibility of all hazards. The difficult course was located in a wooded area with houses, trees, and bushes as distractions (referred to as “ancient”).

The participants were instructed to drive and behave as they would in a real, non-simulated, driving situation. They were asked to be vigilant to street signs and break-down cars emerging on both sides of the road. In addition, they were instructed to react as soon as possible to these stimuli, as well as to potentially hazardous events such as wild boars or colored balls approaching the road, by either pressing the brake or using the indicator or both, as seemed appropriate to them in the respective driving situation. [A total of eight hazardous events occurred during each course, four (two wild boars and two colored balls) from both sides of the road at two different eccentricities.] This task was also practiced in the training session prior to the testing day. Speed was maintained by a cruise control. While depressing the pedal, the car sped up to a constant speed of 70 km/h unless the brake was used. This was implemented to assure comparability of reaction times between age groups, as it is known that older drivers reduce speed as a possible compensatory mechanism (Cantin et al., 2009).

After the examination participants were asked questions regarding their driving history and how they perceived the test and their performance. They were also asked about their experience with video computer games. We included a questionnaire to take into account video game experience as a possible effect on performance. As our group consisted of different ages engaged in different levels of video game experience, we categorized the participants in three groups according to their history. Table 1 shows the grouping criteria for the video game experience. We differentiated virtual media history in PC or video games with animation, such as action games that require quick strategic assessment of situations, and one-dimensional strategic games, such as Tetris or card games. Table 1 displays the division into groups by the time spent on game playing.

**Table 1:** **Grouping criteria for video game experience**.

**Groups**	**Experience with video games**	**Number of participants**	**Mean age**
Group 1	Minimum experience (games without animation/motion perception, such as card games)	39	56.33
Group 2	Medium experience (one to two different kind of video games such as car-racing or flight simulation)	22	36.18
Group 3	Extensive experience (≥2 different kind of video games (see Group 2) regularly played)	12	27.5

### Test parameters

SILAB Software recorded speed and reaction times (use of turn signal or brake). The MATLAB software (MathWorks Company, Natick, MA, USA) was used to analyze the recorded experimental data. Saccades were defined as sections of the gaze trajectory where gaze velocity exceeded 30° /s and gaze amplitude was larger than 1°. Sections between saccades were defined as fixations. Head-movements were defined as movements exceeding 6° /s and an amplitude of more than 3° (Einhäuser et al., 2007). Object fixations were defined as fixation on an object with gaze position maximally 1.24° apart from the object on the *X*-axis and 1.66° on the *Y*-axis. In addition to these measures, the average length of the participant's fixations (mean fixation durations) and the spread of search in the horizontal and vertical meridians (the variance of the fixation locations) were also calculated. The average number of successfully detected targets per participant was recorded.

Simultaneous head- and eye-movements with trajectory in the opposite direction were excluded, as they represent no gain in amplitude of gaze movement. They were subsumed as vestibulo-ocular reflexes (VOR) and excluded from head-movements.

Reaction time was measured in two ways: as a first mode (first detection) we measured reaction time as first detection by either fixation or manual detection: if the participant fixated the object first and responded manually afterward (represented the majority of cases), then we chose the fixation time as reaction time as first detection. If the subject used the turn signal or brake pedal first as an indicator fixating the object afterward, then manual reaction time represented reaction time as first detection. As a second mode (manual reaction), we measured reaction time by manual reaction (brake or turn signal) only.

### Analysis

For statistical analysis linear mixed models (LMM) were used to measure the fixed effects of age, eccentricity of target objects (close vs. distant), course difficulty (easy vs. difficult), and two dummy variables of our three video game experience groups (extensive video game experience and moderate video game experience) and their interactions on reaction times, parameters of saccades (mean fixation duration, mean gaze amplitude, number of saccades, distribution of fixations on the screen) and head-movements with subject as random factor. Effects with a *t*-value larger than ±2 were considered as significant (Kliegl et al., 2010; Ohl et al., 2011). Functions for LMMs were provided by the lme4 package (Bates and Maechler, 2010) in the R environment (R Development Core Team, 2010). Eccentricity of objects was coded as 0 for “close,” 1 for “far” (appearing at 10 m or at 30 m distance to the road). The different types of courses were coded as 0 for the course with a lower level, “field,” and 1 for the higher level of complexity, “ancient.” The baseline group for the dummy variables was minimum video game experience and coded 0 for the variables extensive and moderate video game experience. The dummy variable “moderate video game experience” was coded as 1 for “yes” and 0 for “no,” extensive video game experience and the baseline group. The dummy variable extensive video game experience was coded 1 for “yes,” and 0 for “no” moderate video game experience and the baseline group. Gender was coded 0 for male, 1 for female. Incidence of malaise was coded as 0 for “no” and 1 for “yes.” Age was uniformly distributed in range from 20 to 75 years and centered around the mean (mean = 0.000548, SEM = 0.964175), here referred to as age_c, for use as a covariate in the regression model. Logistic regression was used to calculate the effects of age, eccentricity of target stimuli, course type, and computer game experience on the incidence of simulator sickness. Correlation analyses were obtained by polycor package (Fox, 2007) between age and video game experience. Graphics were obtained by the ggplot2 package (Wickham, 2009) in the R environment (R Development Core Team, 2010).

## Results

### Descriptive statistics

**Figure 1:**
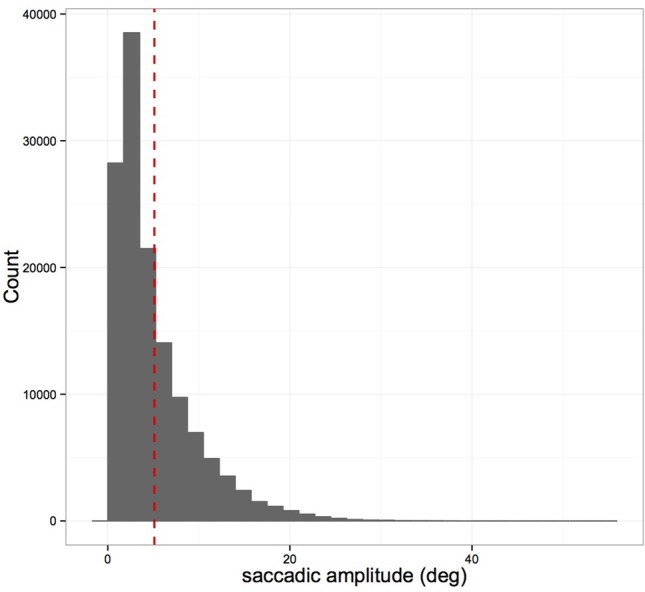
**Distribution of saccade amplitudes (in degree) of 73 subjects**. The majority of saccade amplitudes ranged between 1 and 10°, and below 20°.

**Figure 2:**
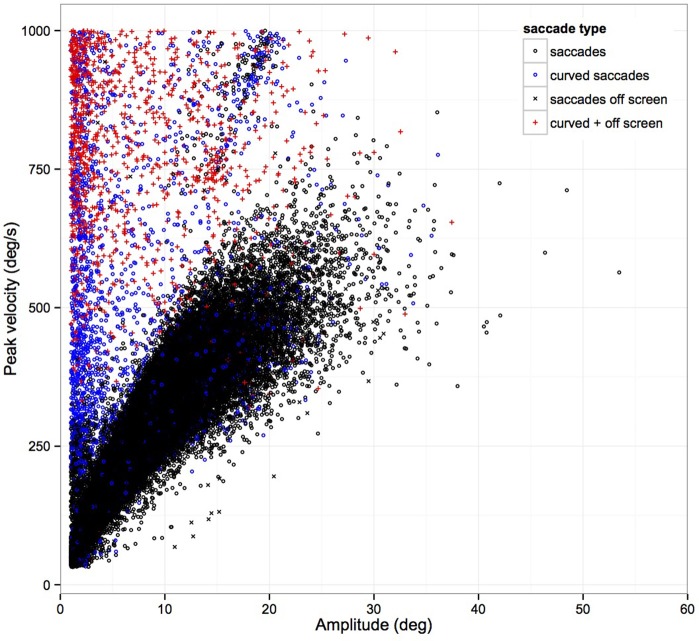
**Main sequence, peak velocity, and amplitudes of 73 subjects**. Peak velocity increases with amplitude of saccades. There are subjects revealing saccades with high velocities and small amplitudes lying outside of the main sequence. These data points represent portions of the gaze trajectory where arc length exceeds cord length. These represent curved saccades or saccades directed downward toward the equipment or vertically along the road (see Descriptive Statistics). Overall these curved saccades presented 2.9% of all saccades. (Saccades are represented with black circles, curved saccades with blue circles, off screen saccades with black X and combined curved and off screen saccades with red crosses.)

**Figure 3:**
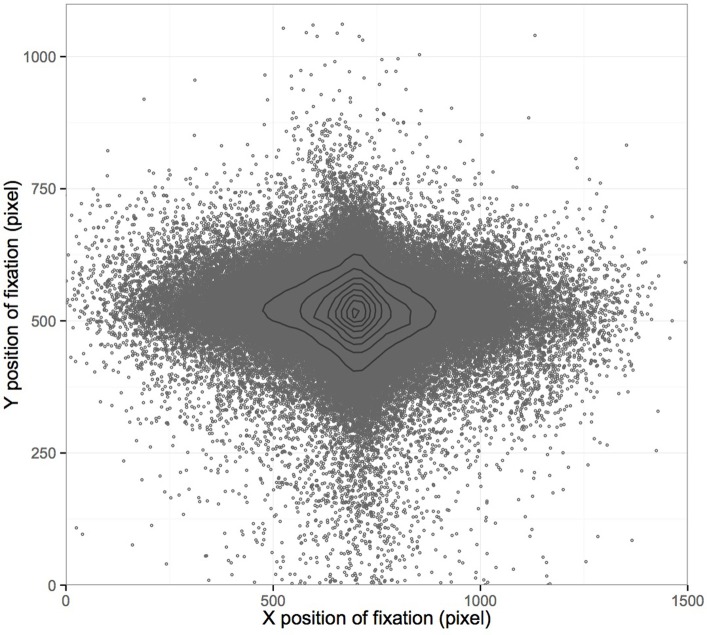
**Distribution of fixations on screen**. Presented are all fixations of 73 subjects and their distribution on the screen, which depicted the driving scenario during the driving task. A density map estimation contours the areas fixated most on screen.

Head-restrained saccades have been characterized by a set of stereotyped relationships, referred to as “the main sequence” (Bahill et al., 1975): the duration and peak velocity of a sac-cade both increase with increasing magnitude of a saccade. These characteristics have been shown to alter with head-unrestrained movements with larger amplitudes above 20° (Freedman, 2008). Figures 1–3 show saccade characteristics in our study. Some subjects performed saccades where the arc length exceeded the cord length by 15°. When examining these saccades in isolation and tracking their course, they represent curved saccades and hence are marked as such in Figure 2 as they are lying out with the main sequence (see Figure 2). Overall these curved saccades presented 2.9% of all saccades. Curved saccades have been described in fixational eye movements (Guerrasio et al., 2010). In addition, these outliers occurred when subjects left the screen with their gaze and eye tracking signal was lost, e.g., to look at the equipment such as the foot pedals. These “off screen saccades” are marked separately in Figure 2. We did exclude vertical saccades with high speed peak velocity > 1000° /s as those likely represent blinks. We did not exclude curved vertical saccades with slower peak velocity directed toward the equipment or along the road as some were directly preceding object fixations, which then would have been missed.

#### Video game experience

To improve our knowledge about the constitution of the different video game level groups, we designed a binomial logistic regression model with video game experience as the dependent variable and age, gender, years of active driving as predictors. The model showed that video game experience was more likely in young and male participants. Table 2 displays the logistic regression model with the video game experience as the dependent variable.

**Table 2:** **Logistic regression model for video game experience (significant effects in bold)**.

	**Estimate**	**SE**	***z*-Value**	**Pr(>|z|)**
Intercept	−2.38666	1.75282	−1.362	0.17332
Gender	−2.74008	1.00837	−2.717	**0.00658**
Age (centered)	−0.18879	0.08548	−2.209	**0.02719**
Driving experience	0.01266	0.02297	0.551	0.58153

**Figure 4:**
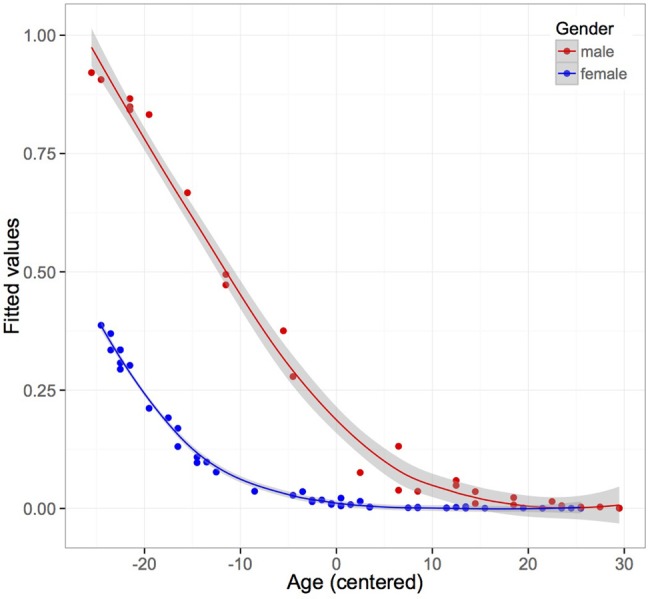
**Fitted values from binomial logistic regression on video game experience**. Male and younger participants reveal more videogame experience than older and female participants (*n* = 73).

With increased age, video game experience reduced significantly, representing a major influence on the data, which needed to be controlled for (see Figure 4).

Furthermore we found a strong negative correlation (*r* = −0.76) between age and the different groups of video game experience, showing that participants with extensive video game experience were younger than people with less or no experience in video gaming.

### Reaction times

All objects were detected and none were missed by any participant. In order to disentangle a collinearity effect between the two main variables of interest, age and levels of video game experience, we calculated two different models for reaction time. In both models, the first with age as main predictor and the second with levels of video game experience as main predictor, regression estimates were not much changed. Therefore we chose to integrate both variables as fixed effects in the same model. We included median manual reaction time as the dependent variable and age, the dummy variables of videogame experience, type of course, target eccentricity as fixed effects as well as their two-way interactions. Subject was treated as random effect. Table 3 displays the LMM statistics for median manual reaction time.

**Table 3:** **Linear mixed model for median manual reaction time (significant effects with *t*-value ±2 in bold)**.

	**Estimate**	**SE**	***t*-Value**
Intercept	1168.8442	106.0349	11.023
Age	2.1077	5.9926	0.352
Eccentricity	1300.1934	102.1377	**12.730**
Type of course	−96.3001	102.1377	−0.943
Age × eccentricity	1.7190	4.6347	0.371
Age × type of course	0.2802	4.6347	0.060
Eccentricity × type of course	956.0274	105.0335	**9.102**
Moderate video game experience	−82.3265	170.3392	−0.483
Extensive video game experience	50.1128	402.1579	0.125
Eccentricity × extensive video game experience	−509.1063	199.4952	−2.552
Type of course × extensive video game experience	196.0442	199.4952	0.983
Eccentricity × moderate video game experience	−269.9960	151.7790	**−1.779**
Type of course × moderate video game experience	71.6985	151.7790	0.472
Age × extensive video game experience	7.1096	20.0873	0.354
Age × moderate video game experience	6.3935	8.4901	0.753
Variance components		S.D.	
Subject		311.22	
Residual		448.70	

**Figure 5:**
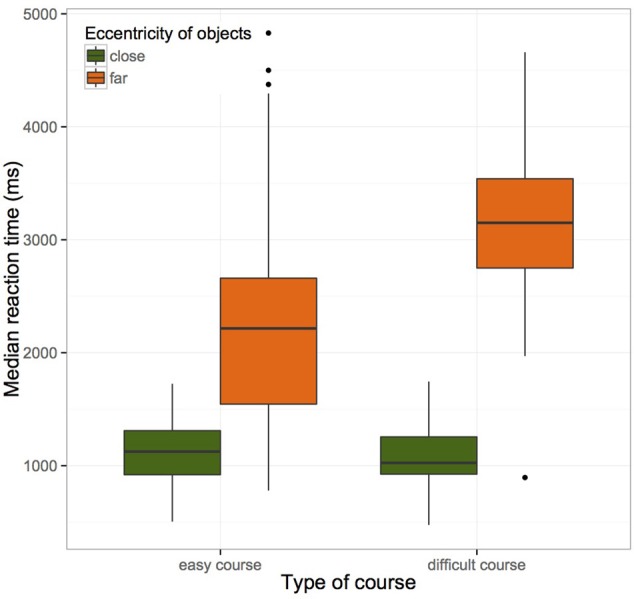
**Boxplots of median manual reaction time (in ms)**. Participants reacted faster to close than to peripheral objects. This effect was amplified by type of course and degree of complexity (easy vs. difficult).

There was a significant main effect of eccentricity on manual reaction time showing that increasing eccentricity of objects is associated with an increase in manual reaction time. Type of course, age, and different levels of video game experience did not show significant main effects, but the interaction of type of course and eccentricity of objects significantly predicted an increase in manual reaction times (see Figure 5). We observed that the interaction of eccentricity of object and extensive video game experience predicted marginal significantly a decrease in manual reaction time. Therefore, manual reaction time to more peripherally located objects decreases with increased video game experience.

In a second step, we examined the reaction time as the first fixation or detection time of objects, and included the same fixed and random effects. Table 4 presents the LMM statistics for the first detection or fixation of objects with both main predictors of interest.

**Table 4:** **Linear mixed model for first detection or fixation of objects (significant effects with *t*-value ±2 in bold)**.

	**Estimate**	**SE**	***t*-Value**
Intercept	874.4402	117.1593	7.464
Age	8.5511	6.2387	1.371
Eccentricity	1243.5934	144.8606	**8.585**
Type of course	−187.0321	144.8606	−1.291
Age × eccentricity	−7.2829	6.5733	−1.108
Age × type of course	−2.4075	6.5733	−0.366
Eccentricity × type of course	852.9680	148.9677	**5.726**
Moderate video game experience	5.4606	187.6357	0.029
Extensive video game experience	230.5355	370.9499	0.621
Eccentricity × extensive video game experience	−514.5448	282.9414	−1.819
Type of course × extensive video game experience	−103.1475	282.9414	−0.365
Eccentricity × moderate video game experience	−280.6056	215.2662	−1.304
Type of course × moderate video game experience	51.5702	215.2662	0.240
Age × extensive video game experience	5.3405	16.6612	0.321
Age × moderate video game experience	0.4521	7.0420	0.064
Variance components		S.D.	
Subject		0.035566	
Residual		636.390273	

And again, increasing eccentricity of objects as well as the interaction of course type and stimulus eccentricity significantly predicted an increase in reaction time. However, neither age nor level of video game experience did affect the first fixation of objects. Thus, our model revealed significant effects on reaction times: the further an object is located in the periphery, the longer the manual reaction time and reaction time by first detection; this effect is amplified by increased difficulty of test course. We found no significant effect of aging alone on reaction times. However, high level of video game experience seems to minimize this effect on manual reaction time, while reaction time by first detection is not influenced by levels of video game experience. We cannot provide a distinct prediction of age or video game experience on reaction times respectively due to their collinearity. Hence, in this study we consider both when examining the participant's performance.

### Exploratory behavior

**Figure 6:**
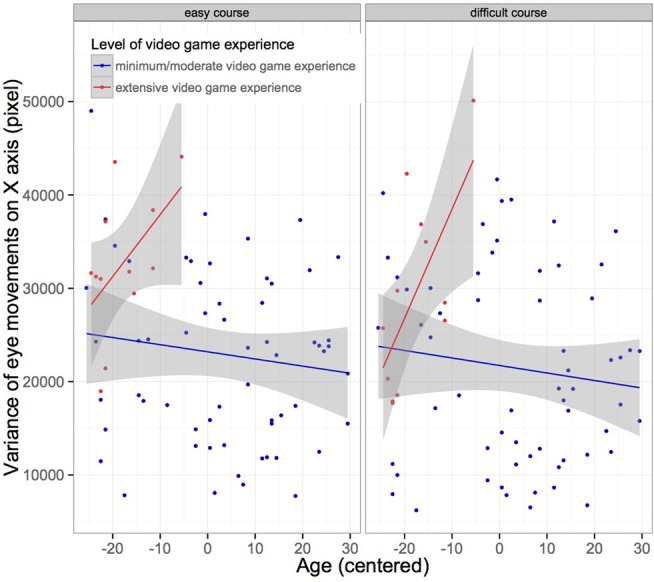
**Measure of mean variance of fixations on horizontal screen (in pixel) shown separately for both type of courses (easy vs. difficult)**. (*n* = 73) (see Materials and Methods). Subjects that had extensive video game experience, playing video action games regularly, showed a wider distribution of fixations on the *X*-axis of the screen. These subjects were of younger age. There were no elderly participants with extensive video game experience.

We included mean fixation duration, mean gaze amplitude, number of saccades, distribution of fixations on the screen and number of head movements as dependent variables in models. Although a strong correlation between age and videogame experience was found, we included the level of videogame experience as predictors in models, as practice of video games might influence the exploratory behavior in participants. Nevertheless, observed effects of video game experience and age alone as well as their interaction effect have to be interpreted carefully (see Figure 4). The video game experience variable was not uniformly distributed across age, because no elder participant had extensive videogame experience in our wide study population (*n* = 73). Therefore we have to consider an extrapolation and overfitting effect of our regression models. Fixed effects were age, type of course, eccentricity of targets, and levels of video game experience, as well as their two-way interactions. Subject was treated as a random effect. There were no significant effects of age alone regarding saccade parameters. However, there was a marginal effect of extensive video game experience on the mean of variance of horizontal fixations on the *X*-axis of the screen, displaying that participants with extensive video game experience covered a wider visual field during the task (see Figure 6). Table 5 displays the LMM statistics for mean variance of horizontal eye movements (on the *X*-axis).

**Table 5:** **Linear mixed model for mean variance of horizontal eye movements on the X-axis (significant effects with *t*-value ±2 in bold)**.

	**Estimate**	**SE**	***t*-Value**
Intercept	23600	2063	11.438
Age (centered)	−110.3	123.9	−0.890
Eccentricity	−3.734e-10	2705	0.000
Type of course	−2898	2705	−1.071
Extensive videogame experience	25280	9090	**2.781**
Moderate videogame experience	−256.6	3326	−0.077
Course × eccentricity	8.256e-11	1030	0.000
Age × course	23.37	45.45	0.514
Age × eccentricity	5.251e-12	45.45	0.000
Age × moderate videogame experience	57.14	202.5	0.282
Eccentricity × moderate videogame experience	1.310e-10	1488	0.000
Course × moderate videogame experience	756.9	1488	0.509
Course × extensive videogame experience	−1219	1956	−0.623
Eccentricity × extensive videogame experience	2.155e-10	1956	0.000
Age × extensive videogame experience	1015	479.2	**2.119**
Variance components		SD	
Subject		8883.6	
Residual		4400.1	

Likewise, the interaction of age and extensive video game experience significantly predicted an increase in the variance of fixations. Thus, the more the drivers have experience in video games and the younger they are, the broader is the field of view they covered with fixations (see Figure 5). In addition, increased video game experience had a significant main effect on mean saccadic amplitude. Table 6 displays the LMM statistics for mean saccadic amplitude.

**Table 6:** **Linear mixed model for mean saccadic amplitude (in bold significant effects with *t*-value ±2)**.

	**Estimate**	**SE**	***t*-Value**
Intercept	4.918	0.2266	21.700
Age (centered)	−0.002116	0.01364	−0.155
Eccentricity	1.892e-13	0.2.822	0.000
Type of course	−0.2650	0.2822	−0.939
Extensive videogame experience	1.874	1.003	**1.869**
Moderate videogame experience	−0.001840	0.3654	−0.005
Course × eccentricity	9.735e-15	0.1075	0.000
Age × course	0.004080	0.004742	0.860
Age × eccentricity	−3.510e-15	0.004742	0.000
Age × moderate videogame experience	0.007429	0.02.237	0.332
Eccentricity × moderate videogame experience	−7.090e-14	0.1553	0.000
Course × moderate videogame experience	0.1134	0.1553	0.730
Course × extensive videogame experience	0.04.884	0.2041	0.239
Eccentricity × extensive videogame experience	−9.707e-14	0.2041	0.000
Age × extensive videogame experience	0.04777	0.05293	0.903
Variance components		SD	
Subject		0.98439	
Residual		0.45913	

Participants with extensive video game experience performed larger mean saccadic amplitudes while driving.

Furthermore, we found a significant main effect of increased age on the number of head movements, demonstrating that with growing age the number of head movements during the task increases. Table 7 displays the LMM statistics for the number of head movements performed.

**Table 7:** **Linear mixed model for the number of head movements (in bold significant effects with *t*-value ±2)**.

	**Estimate**	**SE**	***t*-Value**
Intercept	5.644e + 00	2.264e + 00	2.493
Age (centered)	0.3459	0.1366	**2.532**
Eccentricity	1.916e-14	2.580	0.000
Type of course	−2.050	2.580	−0.794
Extensive videogame experience	12.18	10.08	1.208
Moderate videogame experience	−0.7415	3.651	−0.203
Course × eccentricity	−2.620e-15	0.9826	0.000
Age × course	0.06233	0.04336	1.438
Age × eccentricity	−2.851e-16	0.04336	0.000
Age × moderate videogame experience	−0.4682	0.2253	**−2.079**
Eccentricity × moderate videogame experience	−8.491e-15	1.420	0.000
Course × moderate videogame experience	−0.2510	1.420	−0.177
Course × extensive videogame experience	4.336	1.866	**2.323**
Eccentricity × extensive videogame experience	−3.405e-15	1.866	0.000
Age × extensive videogame experience	0.3286	0.5330	0.616
Variance components		SD	
Subject		9.9611	
Residual		4.1976	

**Figure 7:**
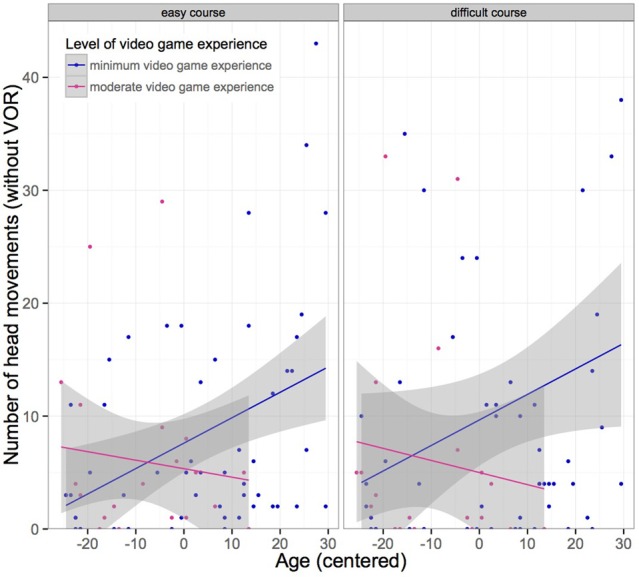
**Measure of the number of head movements without accounting for vestibulo-ocular reflexes (VOR) shown separately for both type of courses (easy vs. difficult)**. Older subjects performed significantly more head movements than younger subjects. However, elderly with moderate video game experience, performed less head movements (*n* = 73).

Also, the interaction of moderate video game experience and age significantly predicted a decrease in head movements. Thus, older participants performed more head movements during the driving task, although if they had moderate video game experience, their frequency of head movements was less (see Figure 7).

### Simulator sickness

In logistic regression analysis we included the incidence of malaise (such as headache, sweating, drowsiness, vertigo as symptoms of simulator sickness) as a dependent variable and age, course type, gender, number of saccades, and video game experience, as well as its two-way interactions, as predictors. We detected a marginally significant trend of an increased number of saccades on incidence of malaise [0.05 (Nagelkerkes), 0.032 (Cox and Snell), χ^2^ = 9.75, *p* = 0.283].

There were no significant effects of age, gender, type of course, and video game experience on the incidence of malaise.

## Discussion

We tested visual exploratory behavior in 73 participants of variable age and its effects on reaction times to hazardous objects relevant to driving safety. Our major interest focused on age-related effects on gaze behavior, information processing, and reaction times in the peripheral vs. central visual field with regard to deterioration of the peripheral visual field. We considered prior experience with video games as a possible influence on test results.

In summary, the results of the present study show that exploratory gaze behavior did not differ with increased age in many saccadic parameters: there were no significant effects of age on mean fixation duration, mean gaze amplitude, and mean number of saccades. However, with increasing age, participants performed more head-movements contributing to the gaze amplitude. While the latter does not change with age, the result suggests a possible compensatory role of head-movements allowing a gaze amplitude large enough to cover the field of view. The role of head-movements as a potential overt attention mechanism in the older populations merits further exploration. It is also unclear whether this could be a mechanism compensating for cognitive decline as suggested by Proudlock et al. (2004). In this respect it is very interesting, that elderly subjects with some video game experience seem to rely less on this potentially compensatory mechanism as they perform less head-movements, raising the question of whether simulation training can improve performance. We demonstrated that subjects with extensive video game experience, i.e., regular animated video game playing, covered a larger field of view with a wide distribution of fixations on screen, as well as slightly larger mean saccadic amplitude and reacted faster manually to peripheral objects, although they did not detect objects faster via fixation. These main findings will be discussed with respect to the deterioration of the UFOV in aging and the role of visual virtual media experience in visual exploratory behavior. We are aware of the fact, that older participants revealed less virtual media knowledge than younger participants, which advocates a careful interpretation of video and age effects on visual exploratory behavior respectively.

The position of the object had a significant effect on reaction times (both manually and via fixation): drivers reacted comparably promptly to close objects without differences in age or video gaming experience. The more distant the objects were located to the subject's gaze position, the longer were reaction times. In previous studies different courses with different visual layout complexity (higher “spatial density”) showed an effect on driving performance (Rogé et al., 2004). Here we also report a significant interaction between object position and type of course. Participants reacted slower manually and fixated later on moving objects in the periphery in the more demanding driving course, including a higher spatial density of visual impressions including bushes, houses, and trees. To clarify whether reaction times to objects of diverse eccentricities change with age, implicating a deterioration of the functional field of view, we examined the reaction times to two different eccentricities with regards to age. Surprisingly, age did neither influence the manual reaction time nor the time it took the driver to fixate the peripheral object after its appearance on the screen. Nevertheless, video game-experienced participants, who were more common among the younger subjects, reacted faster manually to the targets, suggesting either an increase of selective spatial attention or faster manual responses due to better motor abilities (Green and Bavelier, 2003). In previous studies, deterioration of UFOV was supported by experiments relying exclusively on manual reaction (Crundall et al., 2002; Rogé et al., 2004; Jahn et al., 2005). Therefore our results raise the question, whether deterioration of peripheral vision with increased age exists at all when visual attention is measured non-manually, not as a motor-response, by including the measurement of eye movements and when the influence of video game experience is accounted for. Our study cannot address the question of how the peripheral visual field potentially deteriorates gradually providing the classification as general interference or tunnel vision, as we did not provide detailed testing of objects at various eccentricities. Another objection could be directed at the central task, which may be arguably too simple and not highly demanding enough (Williams, 1988) to provide a genuine central task. We did not present a leading car as a central task in our experiment since following a car does not resemble a common driving task in daily life. However we specified a certain speed to provide an equal amount of speed stress on all participants. Therefore we aimed at providing a more naturalistic driving environment which people in rural areas are exposed to.

Furthermore, the results can be interpreted by applying the hypothesis of Pollatsek et al. (2012): if drivers tend to adopt a “habit” perceiving and hence performing driving as a central task, then they react slower manually to peripheral objects because they do not judge them to be acutely relevant. Consequently, they see the objects but do not react immediately due to this habit. Increased video game experience generally seems to resolve this notion of a central task. Our data suggests that it is rather associated with a notion of a general detection task, reacting to all objects as soon as possible. This is of great interest considering a study, which described a general central bias in fixation behavior in subjects viewing a monitor (Tatler, 2007): this central bias was found to become weaker with an active search task, with which fixation distributions were shifted toward the distribution of image features rather than being focused on the center of the screen. Therefore it appears that video gaming experience in our subjects might have altered the understanding of the task, from a driving task to a search task, resulting in different oculomotor behavior with broader visual field and less central bias. Given the fact that the majority of our high level video game players were young drivers, the suspected acquired action video game skills had an effect on the overall performance of the young subjects. This is potentially masking an age effect itself, as in our study these two factors need to be interpreted as linked. In order to further clarify the interaction between age and video game experience, older participants with action video game experience need to be examined, expecting faster manual reaction times to peripheral objects in this group. It will also elucidate the question whether and in what way attentional mechanisms and executive functions differ between video game players and non-video game players.

A critical objection could be raised regarding the recording of reaction times in this study: looking at something does not necessarily imply consciously perceiving it. Hence, reaction times assessed via first fixation (or seldom manual reaction before fixation) may represent an artifact. The error of “seen but not perceived” could jeopardize our interpretation and cannot be ruled out here. Nevertheless, it seems unlikely in the majority of cases that participants could focus via saccades accurately on an object while being unaware of it, especially when a manual reaction follows the fixation.

### Summary and future perspectives

The more demanding the course and the more peripheral the location of the object, the longer were the reaction times, independent of participant age. Deterioration of the useful visual field of view with increased age was not verified in the present study: peripheral objects were detected manually or via fixation at the same speed in all age groups. Interestingly, video game-experienced subjects reacted faster manually to peripheral objects, indicating faster manual responses vs. increased spatial attention and less tendency of perceiving driving “as a central task.” This raised the question, first addressed by Pollatsek et al. (2012), of whether active training, e.g., with driving simulation or video games could improve driving performance, e.g., in the elderly, but also in patients with visual field deficits (Hamel et al., 2012). This exercise could shift attention and encourage faster actual manual response, e.g., with relevance at traffic intersections. It also remains questionable, whether tasks practiced in action video games could be transferred beyond the training task, allowing our subjects with extensive action game experience to better “learn to learn” (Green and Bavelier, 2012). One advantage of a driving simulator testing setting rather than other action gaming options [First person-shooters or Mario Kart DS ® (Boot et al., 2013)] could be, that elderly will likely be more compliant, as driving presents autonomy and a daily useful activity. Whether positive effects could be transferred to real driving or even other cognitive functions, remains unclear.

In the older populations, head-movements increasingly contribute to gaze amplitude. As gaze amplitude does not change with increased age, head-movements seem to assure amplitude size in the elderly. Elderly subjects with some video game experience performed less head movements, again, raising the question of whether simulation training can improve performance.

Head-unrestrained testing in testing sessions with a larger visual field is necessary to uncover the role of head-movements as a compensatory mechanism in the elderly.

Here, we would like to emphasize that the effects of video game experience are more common among the younger subjects, and therefore if not accounted for, it can easily be misinterpreted as underlying age effect. We think it is essential to consider video game experience, specifically with respect to animation setup, in all testing methods using virtual media. Further testing sessions with participants with video game experience more equally distributed among all ages are needed. One hypothesis would be that older participants, experienced in video games, would demonstrate better performance in the driving simulation. The effect of video game experience on exploratory behavior with less central bias and faster reactions to peripheral objects raises the question of whether those subjects drive differently in a real life situation, and whether that would be associated with a lower crash risk in real life driving. This question needs to be addressed in real life driving studies.

### Conflict of interest statement

The authors declare that the research was conducted in the absence of any commercial or financial relationships that could be construed as a potential conflict of interest.
